# A Smartphone-Based Approach to Screening for Sudden Sensorineural Hearing Loss: Cross-Sectional Validity Study

**DOI:** 10.2196/23047

**Published:** 2020-11-11

**Authors:** Heng-Yu Haley Lin, Yuan-Chia Chu, Ying-Hui Lai, Hsiu-Lien Cheng, Feipei Lai, Yen-Fu Cheng, Wen-Huei Liao

**Affiliations:** 1 Department of Medical Education Taipei Veterans General Hospital Taipei Taiwan; 2 Information Management Office Taipei Veterans General Hospital Taipei Taiwan; 3 Big Data Center Taipei Veterans General Hospital Taipei Taiwan; 4 Graduate Institute of Biomedical Electronics & Bioinformatics National Taiwan University Taipei Taiwan; 5 Department of Biomedical Engineering National Yang-Ming University Taipei Taiwan; 6 Department of Otolaryngology-Head and Neck Surgery Taipei Veterans General Hospital Taipei Taiwan; 7 Department of Electrical Engineering National Taiwan University Taipei Taiwan; 8 Department of Computer Science & Information Engineering National Taiwan University Taipei Taiwan; 9 Department of Medical Research Taipei Veterans General Hospital Taipei Taiwan; 10 School of Medicine National Yang-Ming University Taipei Taiwan; 11 Institute of Brain Science National Yang-Ming University Taipei Taiwan

**Keywords:** sudden sensorineural hearing loss, hearing test, telemedicine, mobile apps, pure tone, audiometry

## Abstract

**Background:**

Sudden sensorineural hearing loss (SSNHL) is an otologic emergency that warrants urgent management. Pure-tone audiometry remains the gold standard for definitively diagnosing SSNHL. However, in clinical settings such as primary care practices and urgent care facilities, conventional pure-tone audiometry is often unavailable.

**Objective:**

This study aimed to determine the correlation between hearing outcomes measured by conventional pure-tone audiometry and those measured by the proposed smartphone-based Ear Scale app and determine the diagnostic validity of the hearing scale differences between the two ears as obtained by the Ear Scale app for SSNHL.

**Methods:**

This cross-sectional study included a cohort of 88 participants with possible SSNHL who were referred to an otolaryngology clinic or emergency department at a tertiary medical center in Taipei, Taiwan, between January 2018 and June 2019. All participants underwent hearing assessments with conventional pure-tone audiometry and the proposed smartphone-based Ear Scale app consecutively. The gold standard for diagnosing SSNHL was defined as the pure-tone average (PTA) difference between the two ears being ≥30 dB HL. The hearing results measured by the Ear Scale app were presented as 20 stratified hearing scales. The hearing scale difference between the two ears was estimated to detect SSNHL.

**Results:**

The study sample comprised 88 adults with a mean age of 46 years, and 50% (44/88) were females. PTA measured by conventional pure-tone audiometry was strongly correlated with the hearing scale assessed by the Ear Scale app, with a Pearson correlation coefficient of .88 (95% CI .82-.92). The sensitivity of the 5–hearing scale difference (25 dB HL difference) between the impaired ear and the contralateral ear in diagnosing SSNHL was 95.5% (95% CI 87.5%-99.1%), with a specificity of 66.7% (95% CI 43.0%-85.4%).

**Conclusions:**

Our findings suggest that the proposed smartphone-based Ear Scale app can be useful in the evaluation of SSNHL in clinical settings where conventional pure-tone audiometry is not available.

## Introduction

Sudden sensorineural hearing loss (SSNHL) is an otologic emergency that warrants urgent clinical visits and timely management. SSNHL is commonly defined as a sensorineural hearing loss of 30 or more decibels (dB) over at least 3 consecutive audiometric frequencies occurring within a 72-hour period [1]; it affects approximately 5 to 27 per 100,000 people annually, and its incidence is gradually increasing over time [1-4]. Although SSNHL can occur at any age, the peak incidence occurs among adults aged 45 to 64 years, which is the general age range of working individuals [5]. The typical manifestations of SSNHL include immediate or rapidly progressive hearing loss and, sometimes, hearing loss upon awakening [1]. However, many patients with SSNHL often initially experience only nonspecific symptoms, such as aural fullness or a sensation of a blocked ear, and fail to recognize a loss of hearing, which results in delayed evaluations and treatment [1]. Compounded with the effects of aging and associated symptoms such as dizziness and tinnitus, SSNHL significantly impacts individuals’ general health and quality of life and causes a considerable health care burden [1,6]. Previous studies have identified possible prognostic factors for hearing recovery following SSNHL, including age, severity of hearing loss, duration of hearing loss, and delay in treatment [5,7,8]. As it is a potentially modifiable variable, shortening the time between onset of hearing loss and adequate intervention is a crucial step in improving posttreatment hearing outcomes and minimizing other negative health consequences associated with hearing loss [9-12].

Currently, pure-tone audiometry remains the gold standard for evaluations of SSNHL since it not only reflects the severity of hearing loss but also provides a baseline hearing status for the assessment of recovery [5,8]. Conventional pure-tone audiometry usually requires a standard soundproof booth and calibrated audiometer, is performed by a qualified audiologist, and takes approximately 10 to 20 minutes per patient to perform. Considering the strict requirements regarding equipment and hearing care professionals, the accessibility of timely hearing evaluations using conventional pure-tone audiometry can be limited, especially in primary care settings [13,14]. To address these challenges and optimize the use of hearing health care, the traditional model of hearing screening and health service delivery should be supplemented with more efficient and attainable approaches. For hearing care, a hybrid hearing clinic with both internet-based and in-person services has been implemented in prior research and has revealed high patient satisfaction [15]. In terms of hearing screening, innovative telemedicine tools such as computer-assisted hearing tests [16-19] and mobile phone–based devices [20-23] have been introduced and investigated.

The Hearing Scale Test (HST) is a novel hearing screening tool derived from consecutive hearing screening procedures and used to estimate the current hearing status of each ear; it is based on the concepts of the Landolt C vision test chart [24,25]. With stratified hearing scales that represent various sound levels and four of the main frequencies in speech perception, 0.5 kHz, 1 kHz, 2 kHz, and 4 kHz, the HST not only precisely reflects an individual’s hearing status but also has a computer-based design that enables outcome monitoring and patient surveillance [24]. The HST has demonstrated satisfactory feasibility and accuracy for hearing screening programs in pediatric populations in prior studies [24,25]. A recent study that integrated the HST into a smartphone-based app (Ear Scale) reported remarkable validity for hearing screening among school-aged children [26]. Given the paucity of studies applying innovative mobile phone–based hearing measures to screen for SSNHL, the aim of our study was to determine the correlations of hearing outcomes measured by the proposed smartphone-based hearing screening app with those measured by traditional pure-tone audiometry. We sought to determine the diagnostic validity of the smartphone-based hearing screening approach and, additionally, to explore its role and value in the evaluation of individuals with potential SSNHL.

## Methods

### Study Design and Population

This cross-sectional study was conducted at a tertiary medical center in Taipei, Taiwan, from January 2018 to June 2019. The sample size needed to reach a power of 0.80 was 82. We recruited 88 adults with possible SSNHL who visited either an otolaryngology outpatient clinic or emergency department. The study was approved by the institutional review board of the Taipei Veterans General Hospital (2016-12-004BC). Investigators explained the research objectives and process, and written informed consent was obtained from all patients enrolled. Instructions regarding the screening procedures and operations were provided by the trained examiners prior to each hearing screening test.

### Hearing Measurements

#### Conventional Pure-Tone Audiometric Assessments

Pure-tone audiometry was administered by certified audiologists in the outpatient department. Otoscopy was performed to examine the clearness of the ear canal. Audiometric examinations were performed with a GSI 61 2-channel audiometer (Grason-Stadler Inc) in a soundproof booth. Standard clinical methods (modified Hughson-Westlake methods) were used to obtain pure-tone air conduction thresholds. To assess the reliability of the threshold measures, 1000 Hz was tested twice in each ear; participants with a >10 dB (dB) change between measures were considered unreliable. The pure-tone average (PTA) was calculated using air conduction thresholds at 0.5 kHz, 1 kHz, 2 kHz, and 4 kHz in each ear. Each individual’s pretreatment hearing status measured by conventional pure-tone audiometry was categorized into the following 5 grades according to the modified Siegel criteria for SSNHL [27]: grade 1 (PTA ≤25 dB HL), grade 2 (PTA 26-45 dB HL), grade 3 (PTA 46-75 dB HL), grade 4 (PTA 76-90 dB HL), and grade 5 (PTA >90 dB HL).

#### Smartphone-Based Hearing Screening App

**Figure 1:**
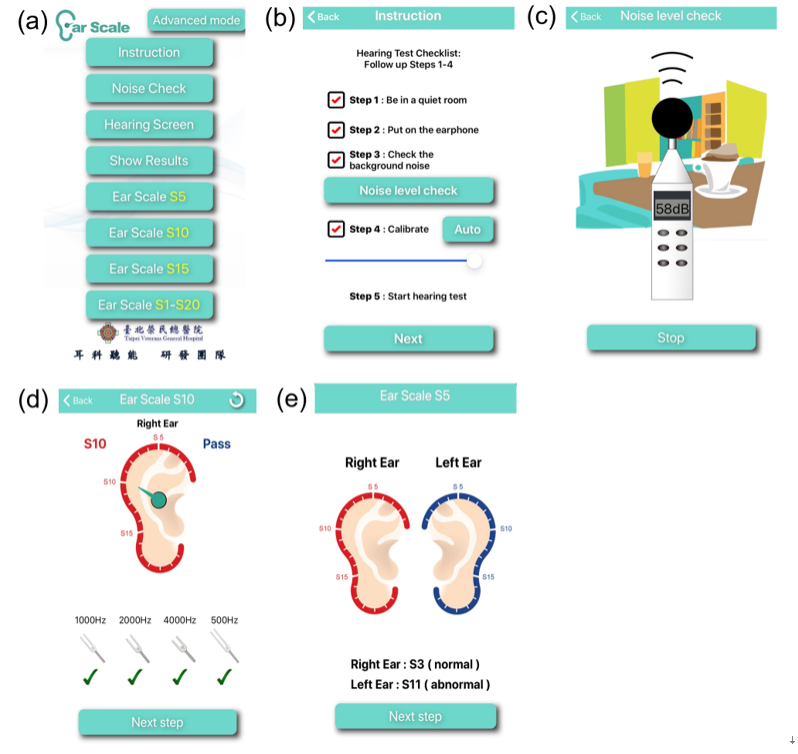
Screenshots of the Ear Scale app instructions for the subjects and the hearing test procedures.

The mobile devices used in this study were the iPhone 7 or iPhone 7 Plus (Apple Inc), with iOS software version 13.3.2. The iOS-based automated Ear Scale app (version 2.0) was integrated with the HST and used to measure the hearing statuses of both ears in the enrolled participants (Figure 1a). The items included in the hearing test checklist were assessed by examiners (Figure 1b). The patients were taught how to wear the headphones and click the response button when they heard the test tones. The headphones used throughout the examination were calibrated for Apple EarPods. The detailed calibration procedures are described in the next section. After the participants put on the headphones correctly, the background noise level was assessed immediately using the built-in function in the Ear Scale app to ensure that the ambient noise was less than 50 A-weighted decibels (Figure 1c). Last, the mobile device and headphones were calibrated and standardized before the HST was started (Figure 1b). The HST incorporated in the Ear Scale app was a novel hearing screening tool developed on the basis of consecutive hearing screening procedures to estimate the current hearing status of each ear [24,25]. The HST measured individuals’ hearing status with respect to stratified hearing scales that represented sound intensity and 4 test frequencies (0.5 kHz, 1 kHz, 2 kHz, and 4 kHz). The adjacent scales differed from one another by 5 dB (Multimedia Appendix 1). The test tones lasted for 1.5 seconds, and the silent intervals lasted for 2 to 3 seconds [26,28]. The Ear Scale app started with hearing scale 5 (S5), which corresponded to 25 dB HL. The four test tones were automatically presented to patients in a fixed order of 1 kHz, 2 kHz, 4 kHz, and 0.5 kHz. The stimulus level of the pure tones descended to the next adjacent hearing scale only if the patient responded correctly to all tones (Figure 1d). The minimum audible hearing scale indicated the lowest pure-tone stimulus level at which the participant responded correctly to all four test tones, was shown at the end of each examination, and was saved to the devices (Figure 1e). The hearing scale difference between the impaired ear and the contralateral ear was determined and used for identifying patients with SSNHL (Figure 1e).

#### iOS Automated Audiometry Calibration

To calibrate the sound output of the iOS mobile devices to a hearing threshold level of zero at various frequencies, we applied the reference equivalent threshold sound pressure levels for Apple EarPods, which were reported in a prior study with consistent output across different EarPod pairs and between the right and left earphones and therefore could be applied to various Apple mobile devices with EarPods [29]. The Knowles Electronics Manikin for Acoustic Research (KEMAR) was initially invented in collaboration with the audiological industry for the use of hearing aid development. KEMAR meets the international standards that are specified by ISO, IEC as well as ANSI. To record the eardrum pressure and evaluate the sound quality, the EarPods were placed in the left and right pinna of a KEMAR manikin, which included a head and torso that had been designed specifically for anthropomorphic testing in the audiologic industry [30]. The microphones of the simulators and the electrical and acoustical measurement systems were calibrated using a 42AA Pistonphone (GRAS Sound & Vibration). The hearing thresholds were determined in an ascending order, as described in ISO 8253-1 [29], with a step size of 1 dB. The initial stimulus level was set to be 10 dB lower than the lowest subject response threshold, which was predetermined by conventional audiometry. Pure-tone stimuli at 0.25 kHz, 0.5 kHz, 1 kHz, 2 kHz, 4 kHz, and 8 kHz were generated on the iOS mobile devices and delivered by the Apple EarPods. All the devices were standardized by setting the user-controllable volume to 100% of the maximum limit. A 2-down, 1-up adaptive staircase procedure was used to determine the final hearing threshold of each subject after 3 reversals [31]. The maximum output difference between the right and left EarPods was less than 1 dB, and the maximum output difference between the devices (iPhone 7 and iPhone 7 Plus) was less than 1.5 dB. The output levels of the EarPods were calibrated in units of dB sound pressure level when the volume of the Apple mobile device was set to the maximum. The output level (dBSPL) of the pure tone at each test frequency was similar to that previously reported [28,29].

### Hearing Screening Procedures

**Figure 2:**
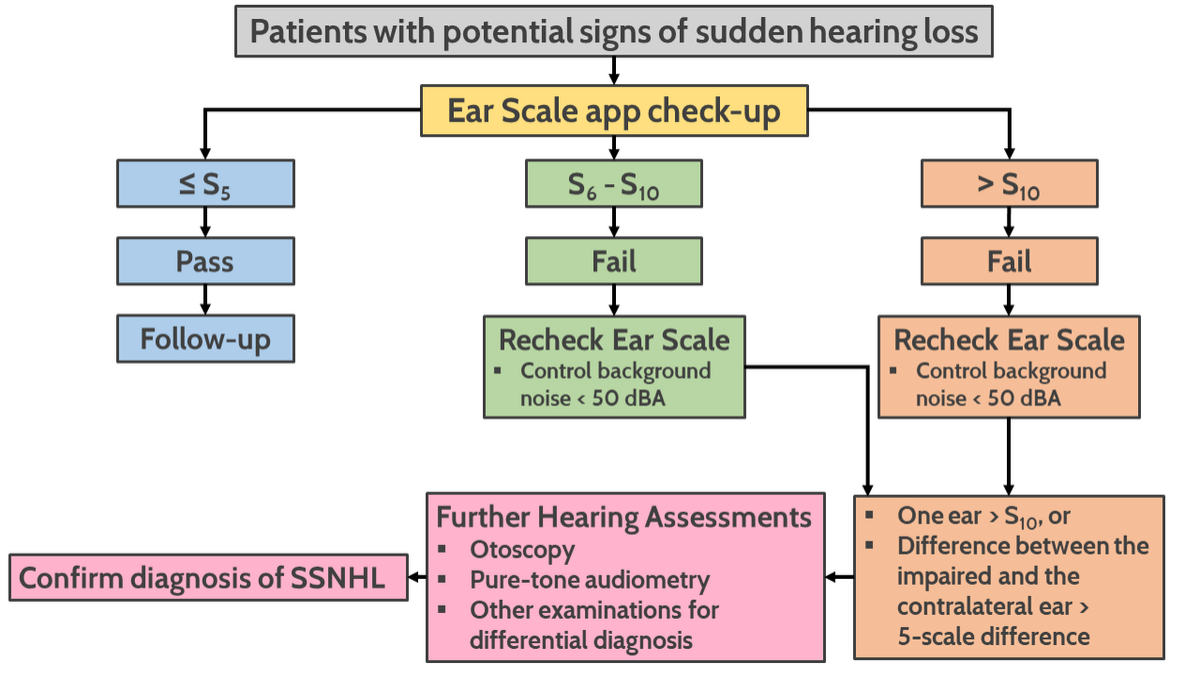
Hearing screening procedures used in this study.

Figure 2 illustrates how the proposed Ear Scale app was used for hearing screening among patients enrolled in this study who had signs of possible sudden hearing loss. Participants underwent the Ear Scale app examination at presentation and were classified into 3 groups (≤S5, S6-S10, >S10) based on their test results. We then arranged comprehensive hearing assessments, including otoscopy, conventional pure-tone audiometry, and other examinations for those who had a hearing scale greater than S10 in one ear or asymmetrical hearing with hearing scale differences greater than 5-scale between the affected ear and the contralateral ear (Figure 2). Participants with bilateral sudden sensorineural hearing loss or conductive hearing loss were excluded from the study population.

### Statistical Analysis

Pearson correlation coefficients were estimated to investigate the correlation between PTA measured by conventional pure-tone audiometry and the hearing scale derived from the Ear Scale app using the HST, as well as the differences in the hearing results between the impaired ear and the contralateral ear of each individual. The corresponding PTA of each hearing scale group was demonstrated using a box plot. Analysis of variance was used to determine the difference in the mean PTA between each scale. Indicators of validity and the predictive value were estimated to determine the diagnostic accuracy of the HST for SSNHL compared with that of the gold standard pure-tone audiometric evaluation. Patients with a PTA difference between the two ears of at least 30 dB within a 72-hour period (ie, the diagnostic gold standard for identifying SSNHL that was used in this study), as assessed by conventional pure-tone audiometry, were considered positive for SSNHL. We then estimated the sensitivity, specificity, positive predictive value, and negative predictive value for diagnosing SSNHL on the basis of 3 hearing scale differences (5-scale difference, 6-scale difference, and 7-scale difference) between the two ears as measured by the Ear Scale app. Sensitivity was defined as the percentage of individuals with true SSNHL (ie, patients with PTA thresholds that met the diagnostic gold standard for the presence of SSNHL included in the American Academy of Otolaryngology-Head and Neck Surgery guidelines [1]) who were correctly identified as having SSNHL by the Ear Scale app. Positive predictive value was defined as the probability of true SSNHL being present among participants who were considered positive for SSNHL by the Ear Scale app. The significance tests for all analyses were 2-sided and included a type I error of .05. The power was set to be 0.80. The statistical software used was Stata 15 (StataCorp LLC).

## Results

### Baseline Characteristics of the Study Sample

This study included 88 adults with possible SSNHL who visited the emergency department or an otolaryngology clinic from January 2018 to June 2019; patients with bilateral or conductive hearing loss were excluded. The mean age of the study cohort was 46 years, and 50% (44/88) were females (Table 1). The average PTA of the cohort included in the analytic cohort was 67.1 dB HL (Table 1). The average hearing scale measured by the Ear Scale app was S17 (ie, 85 dB HL). Regarding the differences in the hearing results between the two ears, the mean PTA difference was 47.6 dB, whereas the average hearing scale difference (obtained from the Ear Scale app) was 9 hearing scales (ie, 45 dB difference; Table 1).

**Table 1:** Baseline characteristics of the study sample (n=88).

Variables		Values
Age in years, mean (SD)		46 (14.7)
Gender (female), n (%)		44 (50)
**Pretreatment hearing grade of worst-hearing ear, n (%)**		
	Grade 1 (PTA^a^ ≤25 dB HL^b^)	7 (8)
	Grade 2 (PTA 26-45 dB HL)	8 (9)
	Grade 3 (PTA 46-75 dB HL)	43 (49)
	Grade 4 (PTA 76-90 dB HL)	16 (18)
	Grade 5 (PTA >90 dB HL)	14 (16)
PTA of worst-hearing ear, dB, mean (SD)		67.1 (24.9)
Average scale of worst-hearing ear, mean (SD)		17 (4.2)
Average PTA difference^c^, dB, mean (SD)		47.6 (25.0)
Average scale difference^d^, mean (SD)		9 (4.4)

^a^PTA: pure-tone average.

^b^dB HL: decibel hearing level.

^c^PTA difference = PTA of impaired ear – PTA of contralateral ear. ^d^Hearing scale difference = hearing scale of impaired ear – hearing scale of contralateral ear.

### Correlation Between the Pure-Tone Average and Hearing Scale

**Figure 3:**
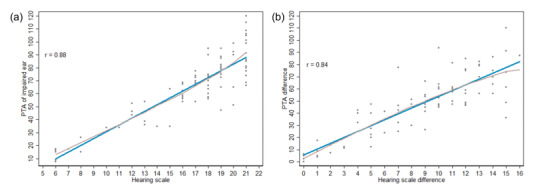
Scatter plots demonstrating the (a) correlation between the pure-tone average (PTA) obtained by pure-tone audiometry (y-axis) and the hearing scale measured by the Ear Scale app (x-axis) and (b) correlation between the PTA differences and hearing scale differences between the impaired and contralateral ears.

The Pearson correlation analyses revealed strong positive correlations between the PTA assessed by pure-tone audiometry and the hearing scale measured by the Ear Scale app as well as between the PTA differences and hearing scale differences between the two ears, with correlation coefficients of .88 (95% CI .82-.92) and .84 (95% CI .77-.90), respectively (Figure 3).

**Figure 4:**
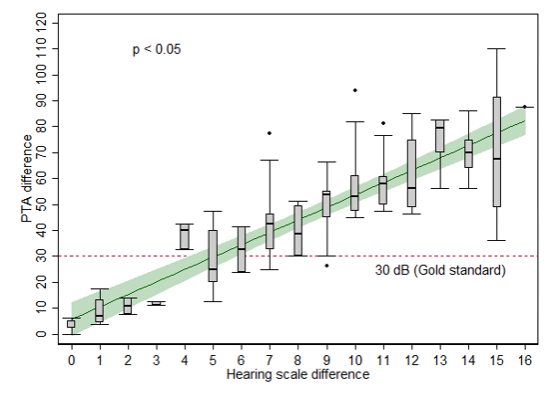
Box plot of the pure-tone average (PTA) difference (y-axis) in relation to the hearing scale difference (x-axis) between the impaired and contralateral ears. The green line depicts the best-fitted mean PTA difference in relation to the hearing scale difference for the linear regression, and the green area represents the 95% confidence interval of the model (*P*<.05, significant differences were found between each hearing scale difference group). The dashed line represents PTA differences of 30 dB (ie, diagnostic gold standard for detecting sudden sensorineural hearing loss in this study).

The association of the PTA and hearing scale differences between the two ears is presented in Figure 4. The mean PTA difference differed significantly across the hearing scale groups (*P*<.05).

### Validity of the Ear Scale App in Diagnosing Sudden Sensorineural Hearing Loss

The diagnostic gold standard for SSNHL used in our study was a PTA difference between the impaired ear and the contralateral ear of ≥30 dB. The indicators of validity of the Ear Scale app and the cutoff values for the hearing scale differences are displayed in Table 2. The 5-scale difference (ie, 25 dB difference) had the highest sensitivity (95.5%, 95% CI 87.5%-99.1%) in diagnosing SSNHL, while the 7-scale difference (ie, 35 dB difference) showed the highest specificity (90.5%, 95% CI 69.6%-98.8%).

**Table 2:** Diagnostic validity of the hearing scale differencea.

Hearing scale difference	Sensitivity, % (95% CI)	Specificity, % (95% CI)	PPV^b^, % (95% CI)	NPV^c^, % (95% CI)
5 hearing scales (25 dB )	95.5 (87.5-99.1)	66.7 (43.0-85.4)	90.1 (80.7-95.9)	82.3 (56.6-96.2)
6 hearing scales (30 dB)	92.5 (83.4-97.5)	85.7 (63.7-97.0)	95.4 (87.1-99.0)	78.3 (56.3-92.5)
7 hearing scales (35 dB)	91.0 (81.5-96.6)	90.5 (69.6-98.8)	96.8 (89.0-99.6)	76.0 (54.9-90.6)

^a^Hearing scale difference = hearing scale of impaired ear – hearing scale of contralateral ear.

^b^PPV: positive predictive value.

^c^NPV: negative predictive value.

## Discussion

### Principal Findings

This study is the first to investigate the validity of a smartphone-based hearing screening app integrated with the novel HST for the assessment of SSNHL. This study confirmed that there is a strong correlation in hearing results between conventional pure-tone audiometry and the proposed smartphone-based Ear Scale app in a cohort of patients with possible SSNHL. The sensitivity of the hearing scale difference between the two ears measured by the Ear Scale app (95.5% for 5-hearing scale difference [25 dB] with a specificity of 66.7%, 92.5% for 6-hearing scale difference [30 dB] with a specificity of 85.7% , and 91.0% for 7-hearing scale difference [35 dB] with a specificity of 90.5%) in diagnosing SSNHL was high, suggesting that the smartphone-based approach can assist in the evaluation of SSNHL, particularly in clinical settings where conventional pure-tone audiometry is not available.

### Comparison With Prior Works

A previous study implemented the proposed Ear Scale app for hearing screening among a pediatric population and reported a strong correlation in the PTA between the app and conventional pure-tone audiometry in a soundproof booth as well as high accuracy in identifying school-aged children with hearing impairment [26]. Notably, Handzel et al [32] used a different smartphone-based app, the uHear hearing test app, for the initial assessment of unilateral SSNHL in 32 patients who had been diagnosed with SSNHL by standard audiometry. Table 3 illustrates the comparison between the gold standard approach (ie, pure-tone audiometry), the uHear app, and the proposed Ear Scale app in this study. The authors observed a sensitivity of 76% with the most stringent gold standard and of 94% with the least stringent criterion when they used the smartphone-based hearing screening tool for diagnosing SSNHL (Table 3) [32]. Our results were consistent with these findings, added to the literature by providing results in a larger sample size and better diagnostic validity and further broadening the population eligible for hearing screening using the hearing scale difference between the impaired ear and the contralateral ear as measured by the Ear Scale app. A significant strength of the proposed method for evaluating SSNHL is that instead of measuring the exact hearing thresholds, we used the hearing scale difference between the two ears to identify SSNHL. A major concern in measuring hearing status using these smartphone- or tablet-based tools is the ambient noise level, since they are not administered in a soundproof booth like conventional pure-tone audiometry. The presence of background noise can negatively affect hearing performance and lead to inaccurate results. This problem was minimized with our approach, as the hearing scale difference between the two ears was used and the influence of ambient noise was therefore canceled out. This unique feature indicates that the implementation of the Ear Scale app can be feasible in noisy environments, thereby broadening its applicability to settings such as urgent care clinics or emergency departments.

**Table 3:** Comparison of key characteristics among different approaches for identifying sudden sensorineural hearing loss.

Diagnostic approach	Author	Audiometric criteria of SSNHL^a^	Role	Sample size, n	Measurement unit	Sensitivity/specificity, %
Conventional pure-tone audiometry	Stachler et al [1]	A decrease in hearing of ≥30 dB, affecting at least 3 consecutive frequencies^b,c^	Gold standard	—^d^	dB HL^e^	—
uHear hearing test app	Handzel et al [31]	Hearing loss of at least 2 hearing grades across 3 or more consecutive frequencies^c,f^	Smartphone-based test	32	Hearing grade	76.0/91.0
Ear Scale app (current study)	Lin et al [10]	Hearing loss of at least 5 hearing scales difference^c^	Smartphone-based test	88	Hearing scale	95.5/66.7

^a^SSNHL: sudden sensorineural hearing loss.

^b^Definition according to the American Academy of Otolaryngology-Head and Neck Surgery guidelines [1].

^c^Hearing loss is defined as related to the opposite ear’s thresholds.

^d^not available.

^e^dB HL: decibel hearing level.

^f^Hearing thresholds are grouped into 6 grades (American Speech-Language-Hearing Association 2012: normal 0-25 dB, mild 26-40 dB HL, moderate 41-55 dB HL, moderately severe 56-70 dB HL, severe 71-90 dB HL, profound >90 dB HL).

### Clinical Implications

Several practice guidelines and reviews have suggested that patients with possible SSNHL undergo a comprehensive clinical workup upon arrival in the clinic, including thorough history taking, relevant physical examinations, and tuning fork tests to differentiate other types of hearing loss from SSNHL, identify nonidiopathic etiologies, and generate differential diagnoses [1,5,33,34]. Although these approaches are important and convenient, they can yield unreliable, even misleading, results [35,36]. Audiometric confirmation is still mandatory for definitively diagnosing SSNHL and should be performed on an emergent basis [1,5]. Conventional pure-tone audiometry remains the preferred method because it accurately distinguishes conductive hearing loss from those of sensorineural origins and establishes frequency-specific hearing thresholds, which are required components of frequently used audiometric criteria for SSNHL [1,5]. Initial audiometric outcomes also provide information essential for predicting prognoses and planning treatments [1]. Given their critical role in the management of SSNHL, audiometric assessments should be performed in accordance with the protocols proposed by the American Speech-Language-Hearing Association and the standards regarding the maximum allowable ambient noise and proper calibration [1,37,38]. In primary care practices (PCPs) or other busy clinical settings, such as urgent care and emergency departments, performing a standard battery of audiology tests can be challenging [13,39]. The high costs of equipment, limited space and time, noisy environments, and shortage of qualified personnel who are capable of conducting the screening and daily health assessments with audiometry are barriers to conventional pure-tone evaluations [13,39,40]. In a study that investigated the procedures performed by general practitioners working in PCPs, less than 20% of clinicians performed audiometry in their practices [41]. Since conventional pure-tone audiometry is mostly unavailable in PCP settings, innovative telehealth approaches have emerged that have been demonstrated as applicable and cost-effective for hearing assessments in PCP-level settings [14,40,42,43]. The Ear Scale app proposed in this study has been shown to be feasible for hearing screenings in the pediatric population [26] and has been shown to have a good level of diagnostic accuracy for SSNHL. We believe that this novel tool, which incorporates the HST and smartphone-based technology, can serve as a point-of-care test for SSNHL at the PCP level because it is affordable, efficient, and requires minimal training to administer. The proposed procedure used in this study (Figure 2) could be the standardized approach when implementing the Ear Scale app in real-world settings. It could therefore assist health care providers in PCP or urgent care settings in making appropriate decisions regarding otolaryngology referrals, reduce the possibility of delayed management, and potentially improve the hearing recovery of patients with SSNHL.

Since the premorbid hearing status is generally unknown among people with possible SSNHL, hearing loss is usually defined on the basis of the difference between the two ears in the thresholds [1]. Based on our results, the PTA differences and hearing scale differences between the two ears are strongly correlated, with a correlation coefficient of .84. The diagnostic validity of three selected hearing scale difference cutoffs is reported in our study. Although all three cutoff values yielded satisfactory sensitivity, we preferred and recommended using the 5-hearing scale difference, as it had the lowest false-negative responses and can serve as the diagnostic standard for SSNHL. There is evidence that patients with untreated/unrecovered SSNHL have more tinnitus and balance problems as well as a poorer long-term quality of life [6,44]. These findings pose significant concerns regarding other negative health consequences associated with hearing loss, including falls [9,45], social isolation [46], depression [47], and incident dementia [10]. In the presence of other common sources of hearing loss, such as presbycusis, the impact of SSNHL is aggravated. In addition, misclassifying diseased cases as nondiseased cases may lead to delayed care among individuals with SSNHL, which is an important prognostic factor because it can be prevented [7,8]. Given that further hearing evaluations of SSNHL, which mainly include standard pure-tone audiometric assessments, are neither invasive nor harmful, minimizing the false-negative rate should be the goal of adequate tools when screening persons with possible SSNHL.

### Limitations

Our results are limited by a high prevalence of SSNHL because the study cohort consisted of patients referred to tertiary academic medical centers. This factor could potentially bias our estimations of the positive and negative predictive values. Additionally, our screening approach may not be applicable for individuals with bilateral hearing loss. Individuals with conductive hearing loss were excluded from the study cohort, and testing such individuals may pose concerns of safety and feasibility. Despite these limitations, our study confirmed that hearing results can be compared between conventional pure-tone audiometry and the proposed Ear Scale app and that the app has sufficient diagnostic validity for SSNHL. To increase the generalizability and ensure the feasibility and safety of this smartphone-based hearing screening approach in PCP or urgent care settings, future studies with prospective designs and larger sample sizes, acceptability surveys among patients and clinicians, and health-economic analyses are needed. Furthermore, the proposed smartphone-based Ear Scale app creates new possibilities in the management of SSNHL at the PCP level since it can serve as a patient surveillance tool, enabling frequent monitoring and treatment adjustments [24,25]. Given the often limited insurance coverage of conventional pure-tone audiometric assessments, implementing a smartphone-based hearing screening approach is a crucial step toward the decentralization of hearing care in the PCP setting, increased accessibility of timely management, and, ultimately, better hearing prognoses in patients with SSNHL.

### Conclusions

This study demonstrated that the hearing results measured by conventional pure-tone audiometry and the proposed smartphone-based Ear Scale app are strongly correlated among patients with possible SSNHL. Our results showed that the hearing scale difference between the two ears, as measured by the Ear Scale app, has a satisfactory level of validity in detecting SSNHL. The results also suggested that this smartphone-based approach may effectively assist the evaluation of SSNHL in clinical settings where conventional pure-tone audiometry is not available.

## Appendix

Multimedia Appendix 1 Sound volume in decibels for each stimulation level examined in the Hearing Scale Test and for different frequencies.

## Abbreviations

dB: decibel
HL: hearing level
HST: Hearing Scale Test
KEMAR: Knowles Electronics Manikin for Acoustic Research
PCP: primary care practice
PTA: pure-tone average
SSNHL: sudden sensorineural hearing loss

## References

[1] Stachler RJ, Chandrasekhar SS, Archer SM, Rosenfeld RM, Schwartz SR, Barrs DM, Brown SR, Fife TD, Ford P, Ganiats TG, Hollingsworth DB, Lewandowski CA, Montano JJ, Saunders JE, Tucci DL, Valente M, Warren BE, Yaremchuk KL, Robertson PJ (2012). Clinical practice guideline: sudden hearing loss. Otolaryngol Head Neck Surg.

[2] Alexander TH, Harris JP (2013). Incidence of sudden sensorineural hearing loss. Otol Neurotol.

[3] Byl FM (1977). Seventy-six cases of presumed sudden hearing loss occurring in 1973: prognosis and incidence. Laryngoscope.

[4] Kuo C, Chung C, Wang C, Chien W, Chen H (2019). Increased incidence in hospitalised patients with sudden sensorineural hearing loss: a 14-year nationwide population-based study. Int J Audiol.

[5] Kuhn M, Heman-Ackah SE, Shaikh JA, Roehm PC (2011). Sudden sensorineural hearing loss: a review of diagnosis, treatment, and prognosis. Trends Amplif.

[6] Härkönen K, Kivekäs I, Rautiainen M, Kotti V, Vasama J (2017). Quality of life and hearing eight years after sudden sensorineural hearing loss. Laryngoscope.

[7] Edizer DT, Çelebi O, Hamit B, Baki A, Yiğit O (2015). Recovery of idiopathic sudden sensorineural hearing loss. J Int Adv Otol.

[8] Cvorović L, Deric D, Probst R, Hegemann S (2008). Prognostic model for predicting hearing recovery in idiopathic sudden sensorineural hearing loss. Otol Neurotol.

[9] Deal JA, Richey Sharrett A, Bandeen-Roche K, Kritchevsky SB, Pompeii LA, Gwen Windham B, Lin FR (2016). Hearing impairment and physical function and falls in the atherosclerosis risk in communities hearing pilot study. J Am Geriatr Soc.

[10] Lin FR, Metter EJ, O'Brien RJ, Resnick SM, Zonderman AB, Ferrucci L (2011). Hearing loss and incident dementia. Arch Neurol.

[11] Deal JA, Sharrett AR, Albert MS, Coresh J, Mosley TH, Knopman D, Wruck LM, Lin FR (2015). Hearing impairment and cognitive decline: a pilot study conducted within the atherosclerosis risk in communities neurocognitive study. Am J Epidemiol.

[12] Reed NS, Altan A, Deal JA, Yeh C, Kravetz AD, Wallhagen M, Lin FR (2019). Trends in health care costs and utilization associated with untreated hearing loss over 10 years. JAMA Otolaryngol Head Neck Surg.

[13] Louw C, Swanepoel DW, Eikelboom RH (2018). Self-reported hearing loss and pure tone audiometry for screening in primary health care clinics. J Prim Care Community Health.

[14] Clark JL, Swanepoel DW (2014). Technology for hearing loss – as we know it, and as we dream it. Disabil Rehabil Assistive Technol.

[15] Ratanjee-Vanmali H, Swanepoel DW, Laplante-Lévesque A (2020). Patient uptake, experience, and satisfaction using web-based and face-to-face hearing health services: process evaluation study. J Med Internet Res.

[16] Masalski M, Kręcicki T (2013). Self-test web-based pure-tone audiometry: validity evaluation and measurement error analysis. J Med Internet Res.

[17] Liao W, Young S, Lien C, Wang S (2011). An audiometer to monitor progressive hearing change in school-aged children. J Med Screen.

[18] Honeth L, Bexelius C, Eriksson M, Sandin S, Litton J, Rosenhall U, Nyrén O, Bagger-Sjöbäck D (2010). An internet-based hearing test for simple audiometry in nonclinical settings: preliminary validation and proof of principle. Otol Neurotol.

[19] Yimtae K, Israsena P, Thanawirattananit P, Seesutas S, Saibua S, Kasemsiri P, Noymai A, Soonrach T (2018). A tablet-based mobile hearing screening system for preschoolers: design and validation study. JMIR Mhealth Uhealth.

[20] Masalski M, Grysiński T, Kręcicki T (2018). Hearing tests based on biologically calibrated mobile devices: comparison with pure-tone audiometry. JMIR Mhealth Uhealth.

[21] Sandström J, Swanepoel DW, Carel MH, Laurent C (2016). Smartphone threshold audiometry in underserved primary health-care contexts. Int J Audiol.

[22] Masalski M, Kipiński L, Grysiński T, Kręcicki T (2016). Hearing tests on mobile devices: evaluation of the reference sound level by means of biological calibration. J Med Internet Res.

[23] Bright T, Pallawela D (2016). Validated smartphone-based apps for ear and hearing assessments: a review. JMIR Rehabil Assist Technol.

[24] Liao W, Lien C, Young S (2010). The Hearing Scale Test for hearing screening of school-age children. Int J Pediatr Otorhinolaryngol.

[25] Liao W, Young S, Tang S (2010). A novel method for quick hearing assessment of children. ICEIE.

[26] Chu Y, Cheng Y, Lai Y, Tsao Y, Tu T, Young ST, Chen T, Chung Y, Lai F, Liao W (2019). A mobile phone-based approach for hearing screening of school-age children: cross-sectional validation study. JMIR Mhealth Uhealth.

[27] Cheng Y, Chu Y, Tu T, Shiao A, Wu S, Liao W (2018). Modified Siegel's criteria for sudden sensorineural hearing loss: reporting recovery outcomes with matched pretreatment hearing grades. J Chin Med Assoc.

[28] Foulad A, Bui P, Djalilian H (2013). Automated audiometry using apple iOS-based application technology. Otolaryngol Head Neck Surg.

[29] Ho C, Li P, Young S (2017). Reference equivalent threshold sound pressure levels for Apple EarPods. J Acoust Soc Am.

[30] Gardner WG, Martin KD (1995). HRTF measurements of a KEMAR. J Acoust Soc Am.

[31] Levitt H (1971). Transformed up-down methods in psychoacoustics. J Acoust Soc Am.

[32] Handzel O, Ben-Ari O, Damian D, Priel MM, Cohen J, Himmelfarb M (2013). Smartphone-based hearing test as an aid in the initial evaluation of unilateral sudden sensorineural hearing loss. Audiol Neurootol.

[33] Jensen EAH, Harmon ED, Smith W (2017). Early identification of idiopathic sudden sensorineural hearing loss. Nurse Pract.

[34] Burgess LP, Frankel SF, Lepore ML, Yim DW (1988). Tuning fork screening for sudden hearing loss. Mil Med.

[35] Stankiewicz JA, Mowry HJ (1979). Clinical accuracy of tuning fork tests. Laryngoscope.

[36] Browning GG, Swan IR, Chew KK (1989). Clinical role of informal tests of hearing. J Laryngol Otol.

[37] American Speech-Language-Hearing Association (1993). Preferred Practice Patterns for the professions of speech-language pathology and audiology. ASHA Suppl.

[38] Frank T (2000). ANSI update: maximum permissible ambient noise levels for audiometric test rooms. Am J Audiol.

[39] Bettger JP, Dolor RJ, Witsell DL, Dubno JR, Pieper CF, Walker AR, Silberberg M, Schulz KA, Majumder P, Juhlin E, Smith SL, Francis HW, Tucci DL (2020). Comparative implementation-effectiveness of three strategies to perform hearing screening among older adults in primary care clinics: study design and protocol. BMC Geriatr.

[40] Louw C, Swanepoel DW, Eikelboom RH, Myburgh HC (2017). Smartphone-based hearing screening at primary health care clinics. Ear Hear.

[41] Flaegel K, Brandt B, Goetz K, Steinhaeuser J (2020). Which procedures are performed by general internists practicing primary care in Germany? A cross-sectional study. BMC Fam Pract.

[42] Swanepoel DW, Myburgh HC, Howe DM, Mahomed F, Eikelboom RH (2014). Smartphone hearing screening with integrated quality control and data management. Int J Audiol.

[43] Yousuf Hussein S, Wet Swanepoel D, Biagio de Jager L, Myburgh HC, Eikelboom RH, Hugo J (2016). Smartphone hearing screening in mHealth assisted community-based primary care. J Telemed Telecare.

[44] Chiossoine-Kerdel JA, Baguley DM, Stoddart RL, Moffat DA (2000). An investigation of the audiologic handicap associated with unilateral sudden sensorineural hearing loss. Am J Otol.

[45] Lin FR, Ferrucci L (2012). Hearing loss and falls among older adults in the United States. Arch Intern Med.

[46] Shukla A, Harper M, Pedersen E, Goman A, Suen JJ, Price C, Applebaum J, Hoyer M, Lin FR, Reed NS (2020). Hearing loss, loneliness, and social isolation: a systematic review. Otolaryngol Head Neck Surg.

[47] Shukla A, Reed N, Armstrong NM, Lin FR, Deal JA, Goman AM (2019). Hearing loss, hearing aid use and depressive symptoms in older adults - findings from the Atherosclerosis Risk in Communities Neurocognitive Study (ARIC-NCS). J Gerontol B Psychol Sci Soc Sci.

