# Supplementary material for: A Smartphone-Based Approach to Screening for Sudden Sensorineural Hearing Loss: Cross-Sectional Validity Study
Source: JMIR Mhealth Uhealth. 2020 Nov 11;8(11):e23047. doi: 10.2196/23047 (PMC7688380; doi:10.2196/23047)
Supplement: Multimedia Appendix 1 [file mhealth_v8i11e23047_app1.docx]

**Multimedia Appendix 1. Sound volume in decibels for each stimulation level examined in the Hearing Scale Test (HST) and for different frequencies.**

|  | Hearing Scale Test (HST) | | | | | | | | | | | | | | | | | | | | | | | |
| --- | --- | --- | --- | --- | --- | --- | --- | --- | --- | --- | --- | --- | --- | --- | --- | --- | --- | --- | --- | --- | --- | --- | --- | --- |
|  | Pretreatment hearing grade ^a,b^ | | | | | | | | | | | | | | | | | | | | | | | |
|  | Grade 1 (PTA $\leq$ 25 dB HL) | | | | | | | Grade 2  (PTA 26$-$45 dB HL) | | | | Grade 3 (PTA 46$-$75 dB HL) | | | | | | | Grade 4 (PTA 76$-$90 dB HL) | | | | Grade 5 (PTA $>$ 90 dB HL) | |
| ^Stimulation level^ | S^b^_1_ | S_2_ | S_3_ | S_4_ | S_5_ | S_6_ | S_7_ | | S_8_ | S_9_ | S_10_ | | S_11_ | S_12_ | S_13_ | S_14_ | S_15_ | S_16_ | | S_17_ | S_18_ | S_19_ | | S_20_ |
| Frequency |  |  |  |  |  |  |  | |  |  |  | |  |  |  |  |  |  | |  |  |  | |  |
| 1, 2 & 4 kHz | 0 | 5 | 10 | 15 | 20 | 25 | 30 | | 35 | 40 | 45 | | 50 | 55 | 60 | 65 | 70 | 75 | | 80 | 85 | 90 | | 95 |
| 0.5 kHz | 5 | 10 | 15 | 20 | 25 | 30 | 35 | | 40 | 45 | 50 | | 55 | 60 | 65 | 70 | 75 | 80 | | 85 | 90 | 95 | | 100 |

Note: PTA = pure-tone average; dB HL = decibel hearing level

a: Based on the modified Siegel criteria

b: Stratified Hearing Scale
